# Repetitive transcranial magnetic stimulation reduces remote apoptotic cell death and inflammation after focal brain injury

**DOI:** 10.1186/s12974-016-0616-5

**Published:** 2016-06-14

**Authors:** Valeria Sasso, Elisa Bisicchia, Laura Latini, Veronica Ghiglieri, Fabrizio Cacace, Valeria Carola, Marco Molinari, Maria Teresa Viscomi

**Affiliations:** Santa Lucia Foundation, I.R.C.C.S., Via del Fosso di Fiorano 64, 00143 Rome, Italy; Dipartimento di Filosofia, Scienze Sociali, Umane e della Formazione, Università degli Studi di Perugia, Perugia, Italy

**Keywords:** Transcranial magnetic stimulation, Inflammation, Apoptosis, Remote degeneration, Glial activation, Neuroprotection

## Abstract

**Background:**

After focal brain injuries occur, in addition to the effects that are attributable to the primary site of damage, the resulting functional impairments depend highly on changes that occur in regions that are remote but functionally connected to the site of injury. Such effects are associated with apoptotic and inflammatory cascades and are considered to be important predictors of outcome. Repetitive transcranial magnetic stimulation (rTMS) is a noninvasive technique that is used to treat various central nervous system (CNS) pathologies and enhance functional recovery after brain damage.

**Objective:**

This study examined the efficacy of rTMS in mitigating remote degeneration and inflammation and in improving functional recovery in a model of focal brain damage.

**Methods:**

Rats that were undergoing hemicerebellectomy (HCb) were treated with an rTMS protocol for 7 days, and neuronal death indices, glial activation, and functional recovery were assessed.

**Results:**

rTMS significantly reduced neuronal death and glial activation in remote regions and improved functional recovery.

**Conclusions:**

Our finding opens up a completely new scenario for exploiting the potential of rTMS as an anti-apoptotic and anti-inflammatory treatment.

**Electronic supplementary material:**

The online version of this article (doi:10.1186/s12974-016-0616-5) contains supplementary material, which is available to authorized users.

## Introduction

The changes that arise at the primary lesion site after a brain focal lesion occurs account for a small fraction of the plastic reorganization that is needed for a good functional outcome [1]. Alterations in regions that are remote to the primary damage are critical [1, 2]. Notably, structural and molecular changes in these remote areas are sustained by many factors, including apoptosis and inflammation [2], for which various pharmacological approaches have been proposed [2].

Repetitive transcranial magnetic stimulation (rTMS) is a noninvasive and easily tolerated method that changes the excitability at the site of stimulation and produces widespread effects at the network level [3, 4], with therapeutic potential for a broad range of neurological and psychiatric disorders [5–10]. Although it has been implemented clinically in many CNS pathologies, the cellular and molecular substrates that underlie the effects of rTMS remain poorly understood [11]. Among the different mechanisms involved, inflammation is one of the possible targets of rTMS effects, although little is analyzed up to now.

The present study addresses the effects of rTMS on remote degenerative mechanisms, such as apoptotic cell death and glial activation, induced by hemicerebellectomy (HCb) [12]. The HCb paradigm is a reliable and effective model for examining remote damage mechanisms and providing a testing ground for novel neuroprotective approaches. In this model, neuronal degeneration is induced by target deprivation and axonal damage of precerebellar neurons [12].

## Methods

### Ethics statement

The experimental protocol was approved by the Italian Ministry of Health (permit number: 444/2015-PR) and conformed to the EU Directive 2010/63/EU for the care and use of laboratory animals. All efforts were made to minimize the number of animals used and their suffering.

### Animals, surgery, and rTMS treatment

Fifty-six male Wistar rats (150–200 g) were used. For surgical procedures, the rats were deeply anesthetized by i.p. injections of xylazine (Rompun; 10 mg/ml; Bayer) and tiletamine and zolazepam (Zoletil 100; 50 mg/ml; Virbac) and the right cerebellar hemisphere was removed as previously described [13]. For the control (Ctrl) group, surgery was interrupted after the dura incision. One hour after surgery, the animals received theta-burst stimulation or sham stimulation (regular coil switched off) by positioning the rat so that the posterior portion of the head was accessible. The coil was held close to the skull between the ears, corresponding to the occipital bone where the wound was sutured, and 10 trains of 50-Hz bursts (3 pulses), repeated at 5 Hz, were applied in 10-s intervals (300 pulses) using a DuoMAG™ XT-100 rTMS through a 70-mm butterfly coil (DEYMED Diagnostic s.r.o., Czech Republic). Stimulus strength was set to 30 % of maximum device output. In the following days, the rats (sham and rTMS) were lightly sedated during treatment. Stimulation was applied daily for 7 days (Additional file 1: Figure S1) by an investigator blind of the experimental group (lesioned vs unlesioned animals).

### Histological procedures and stereological analysis

One hour after the last rTMS treatment, anesthetized animals were perfused transcardially as previously described [13]. Brains were cut using a freezing microtome, and sections were collected in phosphate buffer (PB). One series of brain sections was Nissl-stained [13], while the remaining two series were incubated with a cocktail of primary antibodies including rabbit anti-ionized calcium binding adaptor molecule 1 (Iba-1; 1:400; Wako, Japan), mouse anti-glial fibrillary acidic protein (GFAP; 1:500; Merck Millipore, Italy), mouse anti-neuronal nuclei (NeuN; 1:200; Merck Millipore), and goat anti-cytochrome-c (1:400; Santa Cruz Biotechnology, USA). After washes in PB, sections were incubated with a cocktail of secondary antibodies as previously reported [13]. Images were acquired on a CLSM 700 (Zeiss, Germany). Qualitative and quantitative analyses were limited to the pontine nuclei (Pn) of the experimental side, and the stereological quantification was performed as previously described [13]. Further details are reported in Additional file 2.

### Protein isolation/Western blotting

One hour after the last rTMS treatment, anesthetized rats were sacrificed by decapitation. Pn were isolated, homogenized, and treated as previously described [13]. Samples were incubated with the following primary antibodies: rabbit anti-GFAP (1:2500; Dako, Denmark), rabbit anti-Iba-1 (1:500; Wako, Japan), and mouse anti-cytochrome-c (1:1000; BD Pharmingen, UK). Densities of protein bands in the Western blots were measured, and mean ratios between proteins and β-actin were reported as percentage of control values. The relative levels of immunoreactivity were determined by densitometry using the free software ImageJ (National Institutes of Health, Bethesda, MD, USA). Further details are reported in Additional file 2.

### Quantitative real-time PCR

Quantitative real-time PCR was performed as previously described [13]. The primers used were as follows: rat GFAP F1 (5′-GTCTCGAATGACGCCTCCAC-3′) and rat GFAP R1 (5′-TGTAGCTAGCAAAGCGGTCA-3′); rat Iba-1 F1 (5′-GCAAGGATTTGCAGGGAGGA-3′) and rat Iba-1 R1 (5′-CGTCTTGAAGGCCTCCAGTT-3′); and rat β-actin F1 (5′-ATCCTGACCCTGAAGTACCC-3′) and rat β-actin R1 (5′-AAGGTCTCAAACATGATCTGG-3′). Further details are reported in Additional file 2.

### Functional evaluation

Neurological impairment was evaluated by the neurological severity score (NSS) [13]. NSS is a composite of motor, sensory, reflex, and balance tests, where for each test, one point is awarded for the inability to perform or for the lack of a tested reflex and zero points are awarded for success. A NSS of 18 indicates severe injury, whereas a score of zero signifies healthy, uninjured rats. The NSS was evaluated at 1, 3, 5, and 7 days after damage (5 h after TMS treatment) by an investigator who was blind to the lesioned and unlesioned groups.

### Statistical analyses

All values were expressed as mean ± s.e. All parameters were subjected either to parametric analysis of variance (ANOVA) or to repeated-measure ANOVA. ANOVA was followed, in cases of significance (*P* < 0.05), by post hoc comparisons using Duncan’s test. All quantitative analyses were conducted blind to the animal’s experimental group. All statistical analyses were carried out with the help of Statistica software Version 12.0 (StatSoft, Tulsa, OK, USA).

## Results

**Fig. 1:**
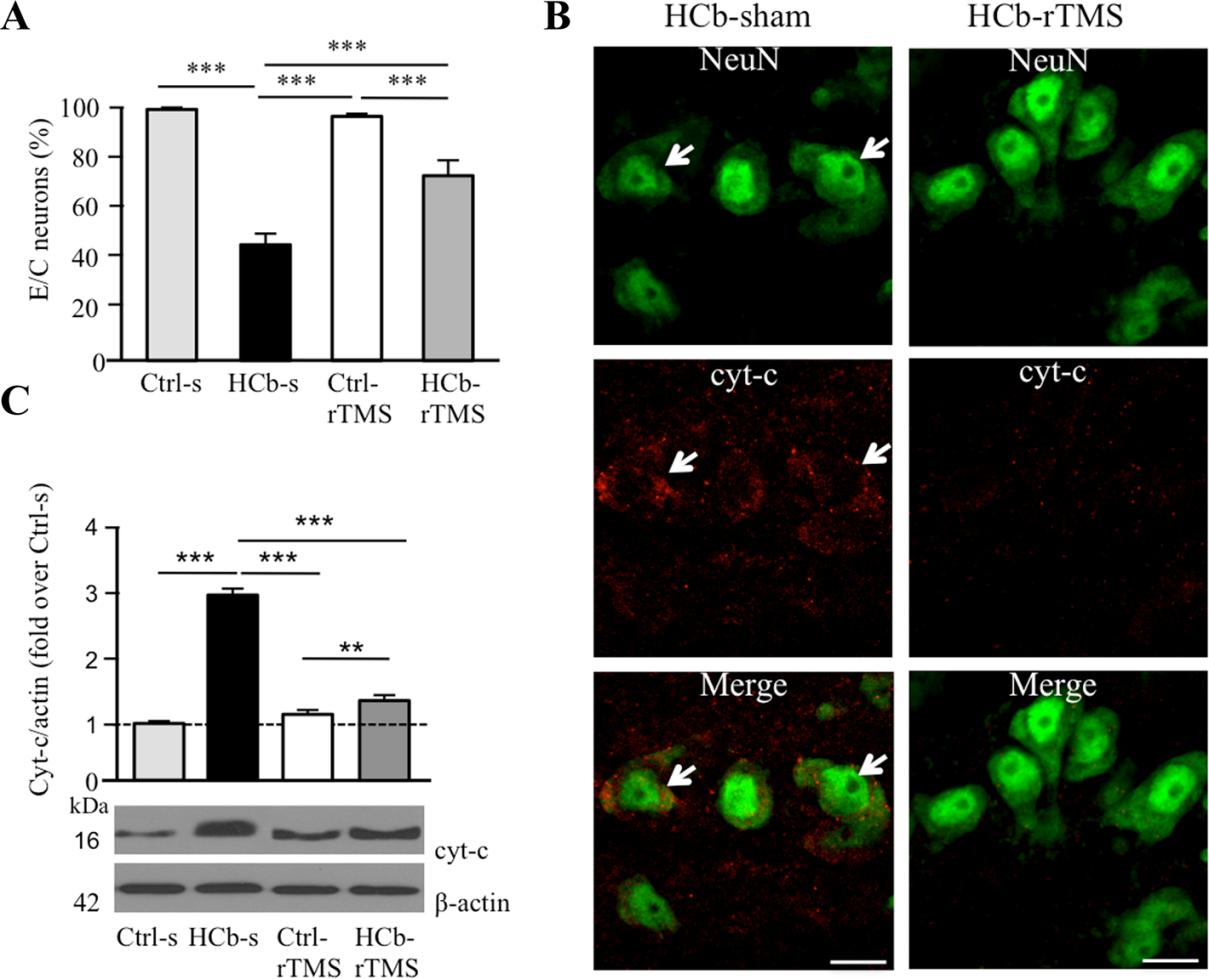
rTMS reduces neuronal death and cytochrome-c release in remote neurons (**a**). Histograms of stereological counts of Nissl-stained neurons in pontine nuclei (Pn) expressed as experimental/Ctrl (E/C) ratio in control-sham (Ctrl-s), HCb-sham (HCb-s), control-rTMS (Ctrl-rTMS), and HCb-rTMS groups. **b** NeuN (*green*) and cytochrome-c (cyt-c; *red*) double-labeling confocal images from pontine nuclei of HCb-sham and HCb-rTMS animals at 7 days after injury showing cyt-c release into the cytosol of neurons (*arrows*). **c** Representative immunoblots and densitometric graphs of cytochrome-c release (cyt-c) in pontine nuclei of Ctrl-s, HCb-s, Ctrl-rTMS, and HCb-rTMS groups. **P < 0.01, ***P < 0.001. *Scale bars*: B = 25 μm

Consistent with our previous results [13–15], HCb induced progressive and severe neuronal death in contralateral Pn 7 days after the lesion (Fig. 1a), associated with increased cytochrome-c (cyt-c) release from damaged mitochondria into the cytosol (Fig. 1b, c).

rTMS treatment significantly reduced HCb-induced neuronal cell death in Pn (group × treatment: *F*[1,16] = 12.130, *P* < 0.003; Fig. 1a). After sham treatment, the Pn population fell to 40 % of prelesional values, whereas after rTMS, over 70 % of Pn neurons remained (Fig. 1a). To investigate the possible effects of rTMS treatment on the apoptotic cascade, we analyzed the mitochondrial cyt-c release in Pn. We observed self-evident differences in cyt-c immunostaining patterns between the HCb-rTMS and HCb-s groups (Fig. 1b). Furthermore, by means of mitochondrial-cytosolic fractionation, we demonstrated that rTMS significantly reduced cyt-c release into the cytosol (group × treatment: *F*[1,12] = 252.417, *P* < 0.001; Fig. 1c). Notably, the rTMS and sham treatments were ineffective in unlesioned rats (Fig. 1a–c).

**Fig. 2:**
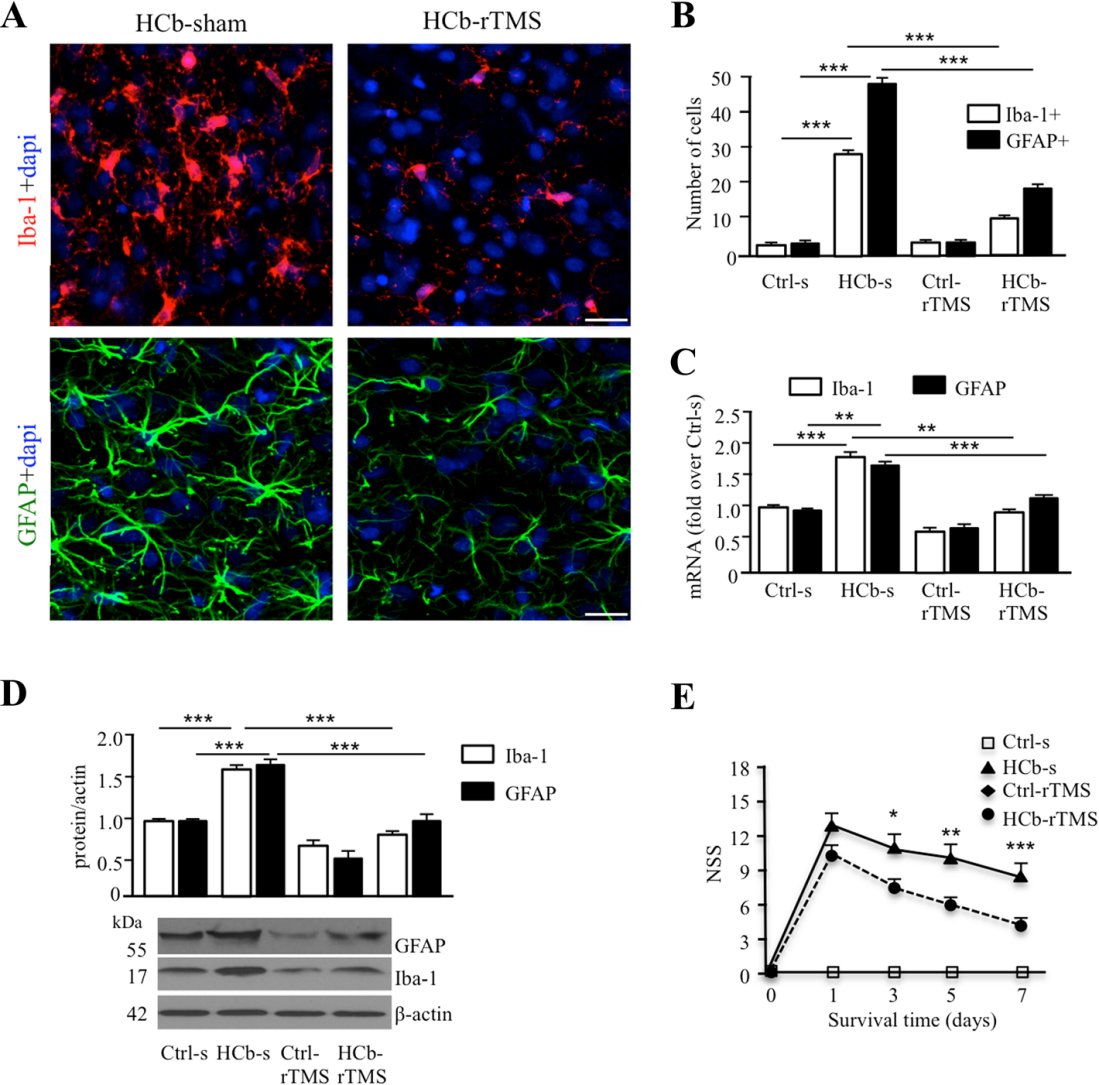
rTMS reduces astrocytes and microglial activation in remote regions and improves functional recovery. **a** Representative confocal microscopy images of Iba-1 positive microglial cells (*red*) and GFAP positive astrocytes (*green*) both counterstained with Dapi (*blue*) in pontine nuclei of HCb-sham and HCb-rTMS groups. **b** Histograms of the number of Iba-1+ microglial cells and GFAP+ astrocytes in pontine nuclei in Ctrl-s, HCb-s, Ctrl-rTMS, and HCb-rTMS groups. **c** Histograms of mRNA expression level of Iba-1 and GFAP in Ctrl-s, HCb-s, Ctrl-rTMS, and HCb-rTMS groups. **d** Representative immunoblots and densitometric graphs of Iba-1 and GFAP protein levels in pontine nuclei of Ctrl-s, HCb-s, Ctrl-rTMS, and HCb-rTMS groups. **e** Time course of neurological recovery (NSS) in the Ctrl-s, HCb-s, Ctrl-rTMS, and HCb-rTMS groups. *P < 0.05, **P < 0.01, ***P < 0.001. *Scale bars*: A = 100 μm

Further, as expected [16], HCb effected intense astrocyte and microglial activation in Pn, as evidenced by the increasing number of GFAP- and Iba-1-positive cells (Fig. 2a, b) and by the upregulation of GFAP and Iba-1 messenger RNA (mRNA) and protein (Fig. 2c, d). rTMS treatment significantly attenuated the HCb-induced glial activation, as demonstrated by the reduction in the total number of GFAP- and Iba-1-positive cells (GFAP, group × treatment: *F*[1,196] = 444.208, *P* < 0.001; Iba-1, group × treatment: *F*[1,196] = 595.584, *P* < 0.001; Fig. 2a, b) and in GFAP and Iba-1 mRNA (GFAP, group: *F*[1,12] = 16.68, *P* < 0.002; GFAP, treatment: *F*[1,12] = 14.85, *P* < 0.002; Iba-1, group: *F*[1,12] = 17.26, *P* < 0.001; Iba-1, treatment: *F*[1,12] = 27.57, *P* < 0.001; Fig. 2c) and protein (GFAP, group: *F*[1,12] = 27.69, *P* < 0.001; GFAP, treatment: *F*[1,12] = 31.44, *P* < 0.001; Iba-1, group × treatment: *F*[1,12] = 12.28, *P* < 0.001; Fig. 2d). No effects were observed in the unlesioned groups (Fig. 2a–d).

Furthermore, rTMS significantly improved functional recovery, as demonstrated by the NSS test (treatment: *F*[1,14] = 9.76, *P* < 0.007; day: *F*[3,42] = 46.91, *P* < 0.001; Fig. 2e), with rTMS inducing greater functional recovery starting from 3 days of treatment (Fig. 2e).

## Discussion

This study demonstrates that rTMS significantly reduces mitochondrial damage, apoptotic neuronal death, and glial activation and supports functional recovery in a rat model of remote damage after focal cerebellar injury.

Here, we showed that rTMS significantly reduced HCb-induced cell death of precerebellar neurons by blocking cyt-c-associated apoptosis. These findings are consistent with earlier reports on the anti-apoptotic effects of rTMS in the perilesional area after TBI [17] and after transient cerebral ischemia [18]. Although remote mechanisms differ substantially from those in perilesional areas after traumatic or ischemic insults [2, 12], our data demonstrate the effectiveness of rTMS in counteracting apoptotic cell death in areas that are distant from the site of damage. Although the efficacy of rTMS in reducing apoptotic cell death in our model is quite specific, further mechanistic studies are required to identify signaling pathways of rTMS effects on precerebellar neurons. In addition to the effects on neurons, our data also showed that glial cells, specifically astrocytes and microglia, responded to rTMS stimulation. In fact, in our model rTMS significantly reduces HCb-induced inflammatory responses, which have been shown to contribute to remote degeneration [2]. At present, there is limited information regarding the response of astrocytes and microglia to rTMS in health and disease [19]. Present data demonstrating the rTMS effects on neuroinflammation, although pointing to a direct effect of TMS on glial cells, do not allow to rule out a direct effect on neurons and their survival. Overall, taking into account the key role of neuron-glia crosstalk in CNS physiology and pathophysiology, the influence of TMS on glial cells is critical to open up novel therapeutic options. However, further studies are needed to clarify the specific effect of TMS on neuron and glial cells as well as on their crosstalk mechanisms to being able to develop TMS approaches for modulating specific cellular responses. In this line, it is worth considering that in our model, as well as in many brain pathologies, plastic responses to injury are not limited to mitochondrial damage or glial activation. We cannot exclude that other factors, in addition to those mentioned, are also sensitive to rTMS. On the other hand, as the clinical significance and positive therapeutic effects of rTMS in a great variety of CNS disorders suggest that they are determined by a combination of multiple factors, we can speculate that, also in our model, rTMS-mediated neuroprotection is a multifactorial process in which many elements play a role. Future research on these mechanisms and factors will be critical for the development of more powerful and reliable TMS therapeutic protocols. In particular, interactions between neurophysiological and cellular/molecular effects of TMS represent a new and intriguing field, which is opening up new lines of research to address neuronal survival and plasticity after CNS insults.

We are aware that the use of a commercial human-sized coil with high-intensity field strengths (≥1 T) might be a limitation of our study [20, 21], rendering dose efficacy or target selectivity requirements unable to be evaluated. However, the selectivity of the effects of rTMS on lesion-induced changes and the patent differences between sham and rTMS treatments support the reliability of our findings. Furthermore, the high sensitivity of the damaged tissue to rTMS is also notable. No changes in any of our parameters were observed in the unlesioned group.

Establishing the link between the sparing of neuronal death in a given population and improvements in functional recovery is always challenging. We cannot exclude that rTMS, especially using so large a coil, might influence outcomes by acting on neural centers that differ from those that we have considered. Plasticity-related changes after rTMS can occur in regions that are functionally connected to the stimulated area and thus contribute to the efficacy of rTMS [22–25]. Despite these cautions, the demonstration of cellular and molecular changes in a key node of the cerebro-cerebellar loop—i.e., the Pn—supports the importance of Pn survival in the recovery.

## Conclusions

In conclusion, although further mechanistic studies are required to identify detailed signal pathways of rTMS effects, our study demonstrates that the effects of TMS are multifactorial and extend beyond the conventional synaptic effects that are usually considered [20, 21]. These effects involve neuronal and glial-dependent mechanisms, both of which have significance in the pathophysiology of various neurological diseases and in the modulation of plastic responses after injury.

These findings open new therapeutic scenarios of paramount importance, demonstrating the potential of rTMS as non-pharmacological approach to counteract apoptosis and inflammation, common players in several CNS diseases, such as stroke, traumatic brain, and spinal cord injuries.

## Abbreviations

ANOVA, analysis of variance; CNS, central nervous system; cyt-c, cytochrome-c; GFAP, glial fibrillary acidic protein; HCb, hemicerebellectomy; Iba-1, ionized calcium binding adaptor molecule 1; NeuN, neuronal nuclei; NSS, neurological severity score; PB, phosphate buffer; Pn, pontine nuclei; rTMS, repetitive transcranial magnetic stimulation; s.e., standard error; T, Tesla; TMS, transcranial magnetic stimulation

## Additional files

Additional file 1: Figure S1. Schematic of the hemicerebellectomy (HCb) model and of the treatment protocol employed in the study. (A) Due to the crossed input-output organization of the cerebellar connections, unilateral lesion of a cerebellar hemisphere induces axonal lesions and subsequent degeneration of the contralateral (experimental side) inferior olive (IO) and pontine nuclei (Pn), with sparing of the IO and Pn on the ipsilateral side (control side). (B) One hour after hemicerebellectomy (HCb; day 0), Ctrl (unlesioned rats) and HCb rats received repetitive transcranial magnetic stimulation (rTMS) or sham stimulation (no coil activation). Stimulation was applied daily for 7 days. DCN: deep cerebellar nuclei; icp: inferior cerebellar peduncle. (TIFF 1031 kb)

Additional file 2: Methods supplementary material: Histological, biochemical, and stereological approaches for pontine nuclei analyses after hemicerebellectomy. (DOCX 19 kb)
