# Supplementary figures and images for: Repetitive transcranial magnetic stimulation reduces remote apoptotic cell death and inflammation after focal brain injury
Source: J Neuroinflammation. 2016 Jun 14;13:150. doi: 10.1186/s12974-016-0616-5 (PMC4908713; doi:10.1186/s12974-016-0616-5)

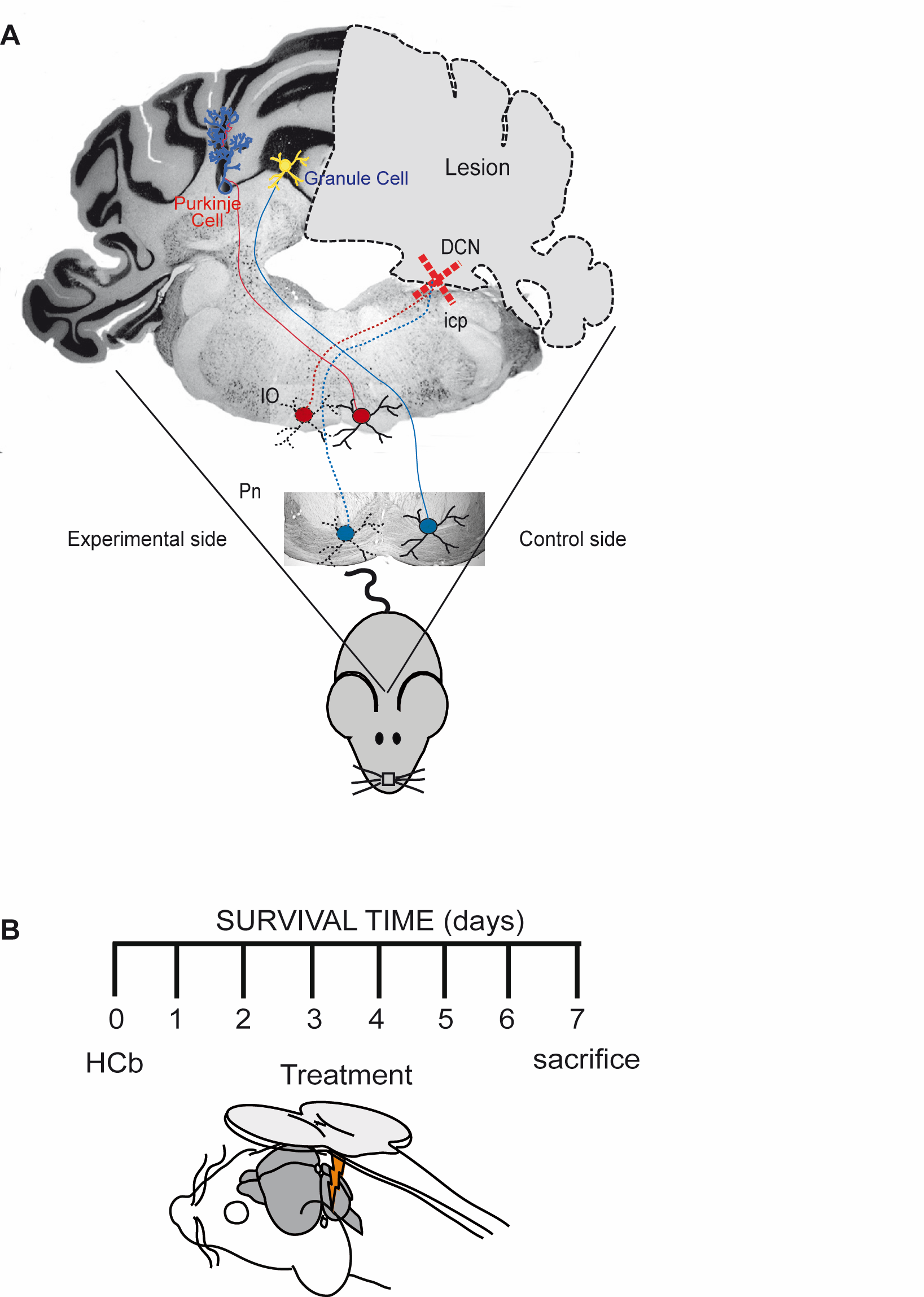

Supplement: Supplementary file 1 — Schematic of the hemicerebellectomy (HCb) model and of the treatment protocol employed in the study. (A) Due to the crossed input-output organization of the cerebellar connections, unilateral lesion of a cerebellar hemisphere induces axonal lesions and subsequent degeneration of the contralateral (experimental side) inferior olive (IO) and pontine nuclei (Pn), with sparing of the IO and Pn on the ipsilateral side (control side). (B) One hour after hemicerebellectomy (HCb; day 0), Ctrl (unlesioned rats) and HCb rats received repetitive transcranial magnetic stimulation (rTMS) or sham stimulation (no coil activation). Stimulation was applied daily for 7 days. DCN: deep cerebellar nuclei; icp: inferior cerebellar peduncle. (TIFF 1031 kb) [file 12974_2016_616_MOESM1_ESM.tiff]
