# Supplementary material for: Repetitive transcranial magnetic stimulation reduces remote apoptotic cell death and inflammation after focal brain injury
Source: J Neuroinflammation. 2016 Jun 14;13:150. doi: 10.1186/s12974-016-0616-5 (PMC4908713; doi:10.1186/s12974-016-0616-5)
Supplement: Supplementary file 2 — Methods supplementary material: Histological, biochemical, and stereological approaches for pontine nuclei analyses after hemicerebellectomy. (DOCX 19 kb) [file 12974_2016_616_MOESM2_ESM.docx]

**Additional file 2**

**Material and methods**

**Histology and immunohistochemistry**

Rats were perfused transcardially with 250 ml of saline followed by 250 ml of 4% paraformaldehyde in a phosphate buffer (PB; 0.1 M; pH 7.4) under anesthesia induced by i.p. injection of the same anesthetic employed below. Each brain was removed immediately, post-fixed in the same fixative for 2 h and, after three washes in PB, transferred to 30% sucrose in PB solution at 4°C until they sank. Brains were cut into four series of 30 μm-thick transverse sections by means of a freezing microtome in three series and were collected and PB.

One series of brain sections were mounted on were mounted on slides and Nissl-counterstained. The remaining two series were incubated d with a cocktail of primary antibodies including rabbit anti-Iba-1 (1:400; Wako, Japan), mouse anti-GFAP (1:500; Millipore), mouse anti-NeuN (1:200; Millipore) and goat anti-cytochrome-c (1:400; Santa Cruz Biotechnology). All primary antibody solutions were prepared in PB and 0.3% Triton X-100 and were incubated overnight. Each incubation step was followed by three, 5-min rinses in PB. Afterwards, sections were incubated 2 h at RT with a cocktail of secondary antibodies, including Alexa Fluor 488 conjugated donkey anti-mouse (1:200; Invitrogen), Alexa Fluor 543 conjugated donkey anti-rabbit (1:200). The specificity of immunohistochemical labeling was confirmed by omission of primary antibodies and use of normal serum instead (negative controls). In order to avoid staining variability, brain sections of the differen experimental groups considered were concomitantly incubated with the same cocktail of primary and secondary antibodies. Sections were rinsed, 4,6-diamidino-2-phenylindole-counter- stained, mounted, coverslipped and then examined using a confocal laser scanning microscope (Zeiss LSM700, Germany). The confocal image acquisitions were performed using consistent settings for laser power and detector gain. Images were exported in TIFF format, contrast and brightness were adjusted, and final plates were composed with Adobe Illustrator CS3 or Corel Draw 9.

**Qualitative and quantitative analyses**

Qualitative and quantitative observations were limited to the pontine nuclei (Pn) of the experimental side that projects to the lesioned hemicerebellum.

To assess the extent of cell loss in PN following HCb, stereological cell count Nissl-stained neurons identified by a clear nuclear profile, was performed. To better appreciate the effects of HCb on pontine neuronal loss, we related the number of surviving PN neurons to the number of neurons present in the PN of unlesioned animals (CTRL), here indicated as E/C ratio.

Using the Stereo Investigator System (MicroBrightField Europe e.K., Magdeburg, Germany), an optical fractionator stereological design was applied to obtain unbiased estimates of total Nissl-stained neurons. A three-dimensional optical dissector counting probe (x, y, z dimension of 30 x 30 x 10 mm, respectively) was applied. The PN was outlined using the 4x objective, while the 100x oil immersion objective was used for marking the neuronal cells. Total number of PN was estimated according to the formula given below:

N = SQ x 1/ssf x 1/asf x 1/tsf

where SQ represents the total number of neurons counted in all optically sampled fields of the PN, ssf is the section sampling fraction, asf is the area sampling fraction, and tsf is the thickness sampling fraction.

Quantitative analysis of GFAP+ and Iba+ cells was performed off- line on confocal images acquired through the 20× objective at the 0.07 zoom factor. All GFAP or Iba-1 labeled cells in three digital squared frames (250 μm per side) in five sections, regularly spaced throughout the caudo-rostral extent of the nucleus, were counted. All quantitative analyses were conducted blind to the animal's experimental group.

**Western blotting**

After PN isolation, tissues were homogenized in in lysis buffer (50 mM Tris–HCl (pH 7.5), 320 mM sucrose, 10% glycerol, 50 mM NaCl, 1% Triton X-100, 1 mM PMSF, with protease inhibitor cocktail (Sigma, #P8340)), incubated on ice for 30 min and centrifuged at 13,000×g for 20 min. The total protein content of resulting supernatant was determined. Proteins were applied to SDS-PAGE and electroblotted on a PVDF membrane. Immunoblotting analysis was performed using a chemiluminescence detection kit. The relative levels of immunoreactivity were determined by densitometry using the software ImageQuant 5.0.

Samples were incubated with the following primary antibodies: rabbit anti-GFAP (1:2500; Dako), rabbit anti-Iba-1 (1:500; Wako), mouse anti-cytochrome-c (1:1000; BD Pharmingen, UK). Densities of protein bands in the Western blots were measured, and mean ratios between proteins and β-actin were reported as percentage of control values.

**Mitochondrial and cytosolic fraction**

Pontine nuclei were homogenized in Buffer A (320 mM sucrose, 1 mM EDTA, 50 mM TRIS-HCl pH 7.4, 1 mM DTT, 1 mM PMSF), with protease inhibitor cocktail (Sigma, P8340) by 30 strokes with a glass Pyrex micro homogenizer. The homogenate was centrifuged at 1,000 × g for 10 min and the resulting supernatant was centrifuged at 10,000 × g for 20 min to obtain the mitochondrial pellet and the supernatant. The mitochondria-containing pellet was washed three times with Buffer B (250 mM sucrose, 1 mM EGTA–Sigma, E3889, 10 mM TRIS-HCl pH 7.4) by centrifugation for 10 min at 10,000 × g. The supernatant was centrifuged at 100,000 × g for 1 h to generate the cytosolic fraction.

**Quantitative real-time PCR**

RNA was extracted from Pontine nuclei (PN) using TRIzol reagent (Invitrogen). Any remaining genomic DNA was eliminated with RNase-Free DNase Set (Qiagen, Germantown, MD, USA) according to the manufacture’s instruction. 1 µg of total RNA was used for RT reaction by using the SuperScript VILOTM cDNA Synthesis Kit (Invitrogene). The following RT-PCR program was used: 25°C for 10 min, 42°C for 60 min, 85°C for 5 min. The expression of the different primers was assessed by quantitative RT-PCR (qRT-PCR), using SensiMixTM SYBR Kit (Bioline) as fluorescent dye to monitor cDNA amplification. The following PCR program was used: 95°C for 10 min, 40 cycles at 95°C for 5 s and 60°C for 100 s. The primers used were: rat GFAP F1 (5’-GTCTCGAATGACGCCTCCAC-3’) and rat GFAP R1 (5’-TGTAGCTAGCAAAGCGGTCA-3’); rat Iba-1 F1 (5’- GCAAGGATTTGCAGGGAGGA-3’) and rat Iba-1 R1 (5’- CGTCTTGAAGGCCTCCAGTT-3’); rat β-actin F1 (5′- ATCCTGACCCTGAAGTACCC-3′) and rat β-actin R1 (5′- AAGGTCTCAAACATGATCTGG-3′). One microliter of the first-strand cDNA product was used for amplification in triplicate. A twenty-five-microliter reaction solution contained 12.5 μl of Platinum SYBR Green qPCR SuperMix-UDG and 10 pmol of each primer. The following PCR program was used: 95°C for 10 min, 40 amplification cycles at 95°C for 30 s, 56°C for 30 s, and 72°C for 30 s. Serial dilutions (10, 100, 1,000, 10,000, and 100,000-fold) of each studied transcript were used to determine the amplification efficiency of

each target and housekeeping gene. Quantitative RT-PCR (qRT-PCR) analyses of mRNA levels were performed comparing mRNA extracts from unlesioned and hemicerebellectomized rats using the 2−ΔΔCT method Viscomi et al. [6]. In the present study, data are presented as the fold-change in target gene expression normalized to the internal control gene and relative to the normal tissue control. The average threshold cycle (CT) was calculated for both the target genes and β- actin, and ΔCT was determined as [the mean of the triplicate CT values for the target gene] − [the mean of the triplicate CT values for β-actin]. ΔΔCT represented the difference between the paired tissue samples, as calculated by the formula ΔΔCT 0 (ΔCT of HCb-Eth − ΔCT of CTRL). The n-fold differential expression of the target gene of an HCb sample compared with the normal counterpart was expressed as 2−ΔΔCT.
